# Risk Stratification after Biochemical Failure following Curative Treatment of Locally Advanced Prostate Cancer: Data from the TROG 96.01 Trial

**DOI:** 10.1155/2012/814724

**Published:** 2012-12-24

**Authors:** Allison Steigler, James W. Denham, David S. Lamb, Nigel A. Spry, David Joseph, John Matthews, Chris Atkinson, Sandra Turner, John North, David Christie, Keen-Hun Tai, Chris Wynne

**Affiliations:** ^1^School of Medicine and Public Health, The University of Newcastle, University Drive, Callaghan, NSW 2308, Australia; ^2^Department of Radiation Oncology, Calvary Mater Newcastle, Locked Bag 7, Hunter Region Mail Centre, NSW 2310, Australia; ^3^Department of Radiation Oncology, Wellington Cancer Centre, Riddiford Street, Newtown, Wellington 6021, New Zealand; ^4^Department of Radiation Oncology, Sir Charles Gairdner Hospital, Hospital Avenue, Nedlands, Perth, WA 6009, Australia; ^5^Department of Radiation Oncology, Auckland Hospital, 2 Park Road, Grafton, Auckland 1023, New Zealand; ^6^Department of Radiation Oncology, St George's Cancer Care Centre, 131 Leinster Road, Strowan, Christchurch 8014, New Zealand; ^7^Department of Radiation Oncology, Westmead Hospital, Corner Hawkesbury Road and Darcy Road, Westmead, NSW 2145, Australia; ^8^Department of Radiation Oncology, Dunedin Hospital, 201 Great King Street, Dunedin 9054, New Zealand; ^9^Department of Radiation Oncology, Premion, Inland Drive, Tugun, QLD 4224, Australia; ^10^Department of Radiation Oncology, Peter MacCallum Cancer Centre, St Andrews Place, East Melbourne, VIC 3002, Australia

## Abstract

* Purpose*. Survival following biochemical failure is highly variable. Using a randomized trial dataset, we sought to define a risk stratification scheme in men with locally advanced prostate cancer (LAPC). *Methods*. The TROG 96.01 trial randomized 802 men with LAPC to radiation ± neoadjuvant androgen suppression therapy (AST) between 1996 and 2000. Ten-year follow-up data was used to develop three-tier post-biochemical failure risk stratification schemes based on cutpoints of time to biochemical failure (TTBF) and PSA doubling time (PSADT). Schemes were evaluated in univariable, competing risk models for prostate cancer-specific mortality. The performance was assessed by c-indices and internally validated by the simple bootstrap method. Performance rankings were compared in sensitivity analyses using multivariable models and variations in PSADT calculation. *Results*. 485 men developed biochemical failure. c-indices ranged between 0.630 and 0.730. The most discriminatory scheme had a high risk category defined by PSADT < 4 months or TTBF < 1 year and low risk category by PSADT > 9 months or TTBF > 3 years. *Conclusion*. TTBF and PSADT can be combined to define risk stratification schemes after biochemical failure in men with LAPC treated with short-term AST and radiotherapy. External validation, particularly in long-term AST and radiotherapy datasets, is necessary.

## 1. Introduction

Biochemical failure is a very common problem in the treatment of prostate cancer. Klotz has estimated that approximately 40% of men treated curatively by prostatectomy or radiotherapy will develop biochemical failure [1]. In the United States, the figure is expected to be at least 60,000 per annum [2]. Outcomes following biochemical failure are known to be highly variable. Clinical signs of local or distant progression can follow within months but may take years to become evident, and five year prostate cancer-specific survival probability has been shown to vary between 35% and 100% [3]. An important breakthrough in the management of prostate cancer is the emergence of effective new options for the treatment of castrate-resistant prostate cancer (CRPC) [4]. Presently these options are routinely withheld until castration-resistant tumour growth develops; however, many clinicians now believe that earlier intervention may be beneficial. The identification of subgroups of men with unfavourable outcomes after biochemical failure, needing immediate treatment and/or new therapeutic agents, is therefore a high priority in clinical prostate cancer research.

Prognostic data can provide assistance to clinical management practices if presented in the form of a nomogram or risk stratification scheme. Nomograms are of value to individual patients and their clinicians when determining the need for interventions. Risk stratification schemes, however, are more valuable in identifying patient subgroups who would benefit from participation in trials of new therapeutic approaches and for stratification purposes in these trials.

With trials of the effective new CRPC agents in mind, we sought to develop a risk stratification scheme to predict the outcome following biochemical failure using data from the TROG 96.01 randomized controlled trial which addressed the value of neoadjuvant androgen deprivation prior to and during radiotherapy for locally advanced prostate cancer. Earlier reports from the TROG 96.01 trial showed that PSA doubling time (PSADT) and time to biochemical failure (TTBF) were independent and highly prognostic variables after biochemical failure [16] and at various cutpoints were successful surrogate candidates for prostate cancer-specific mortality (PCSM) [17]. Other reports have also affirmed their prognostic value [8, 10, 13, 15, 18–31]. Using combinations of PSADT and TTBF, we therefore explored the predictive accuracy of different risk stratification schemes in our trial dataset with minimum 10-year follow-up data from randomization. So far as we are aware, this paper describes the first risk stratification scheme for men with locally advanced prostate cancer who experience biochemical failure after curative radiotherapy with or without adjuvant androgen suppression therapy.

## 2. Materials and Methods

### 2.1. Trial Design

The trial was opened in 1996 following institutional ethical approval at 19 sites across Australia and New Zealand. All patients provided written informed consent. 802 eligible patients receiving radiotherapy (66 Gy delivered using 33 daily fractions of 2 Gy) for locally advanced prostate cancer were randomized to receive 0, 3, or 6 months maximal androgen suppression therapy (AST) prior to and during radiation. This comprised goserelin 3.6 mg (AstraZeneca Pty Ltd., Sydney, Australia) given subcutaneously every month and flutamide 250 mg (Schering-Plough Pty Ltd., Sydney, Australia) given orally three times daily. AST commenced 2 months prior to radiation in the 3-month arm and 5 months prior to radiation in the 6-month arm. Patients were stratified by age (<70 versus 70–80 versus >80 years), tumour stage (T2b and T2c versus T3 and T4), tumor differentiation (well versus moderate versus poor), and initial PSA level (<20 versus ≥20 *μ*g/L). Detailed study design is described elsewhere [32]. The type and timing of secondary therapeutic intervention (STI) was recorded and serial PSA measures were mandated up to death.

Biochemical failure (BF) was defined according to the Phoenix definition (time from end of radiotherapy to a PSA rise of ≥2 *μ*g/L above the post-treatment nadir value) [33]. PSADT estimates were based on PSA values from immediately prior and up to 6 months after biochemical failure and were derived from the slope of the regression line of best fit through the log transformed PSA values selected. Errors in estimating time to biochemical failure and PSADT in our dataset were described previously in Lancet Oncology 2008 [17]. In this dataset, TTBF and PSADT were correlated, but not strongly so. Both variables were independently prognostic for PCSM (data not shown).

### 2.2. Endpoints

Endpoints used in this paper were the cumulative incidences of PCSM, distant progression, and STI from BF. PCSM occurred at the time of death due to prostate cancer (attribution of cause validated in Lancet Oncology 2011 [34]). Distant progression occurred on the date when the first evidence (clinical, radiological, or isotopic bone scan) of metastatic disease in lymph nodes, skeleton, or other site outside of the prostatic region became available. STI occurred when the first type of secondary therapy commenced.

### 2.3. Statistical Methods

The analysis group consisted of 485 subjects who experienced BF prior to clinical failure or STI. Three-tier post-BF risk categorization (BFRC) schemes based on low, intermediate, and high risk groups were derived in two stages: (1) 12 “cut point range-finding” schemes were identified and evaluated using combinations of TTBF and PSADT cutpoints regularly cited in the literature as being predictive of outcome following BF and (2) new “candidate” BFRC schemes were derived based on the most prognostic ranges identified in the range-finding schemes. To ensure that they were unique and consisted of sizeable risk strata, candidate schemes had to satisfy three criteria: at least three months separation between the high and low risk PSADT; at least one year separation between the high and low risk TTBF; and a minimum of 20% of patients in each risk stratum. All BFRC schemes were evaluated in unadjusted regression models for PCSM from BF using the method of Fine and Gray [35]. The performance of each BFRC scheme was assessed by calculating the Harrell's concordance c-index [36]. The c-index is a measure of predictive discrimination and is defined as the proportion of patient pairs in which predictions and outcomes are concordant. A c-index of 0.5 indicates no predictive ability and 1.0 indicates perfect predictive accuracy. Differences between c-indices were computed using a paired Student's *t*-test. The most predictive BFRC model (i.e., with the highest c-index) was identified and internally validated by the simple bootstrap method [36] using 200 replications with replacement to obtain an optimism-corrected performance estimate.

Sensitivity analyses were undertaken to compare the performance rankings of the candidate schemes in a range of adjusted PCSM models. The first model adjusted for trial arm (0 versus 3 versus 6 months maximal AST) as duration of androgen suppression could influence outcome after BF. The second model adjusted for baseline factors as well as trial arm because these could determine the aggression of the relapse process. Additional covariates included age at BF (continuous, years), pretreatment PSA (<10 versus ≥10 and <20 versus ≥20 *μ*g/L), Gleason score (2–6 versus 7 versus 8–10), and tumour stage (T2b versus T2c versus T3 and T4). The third model also adjusted for STI as an ordinal time-dependent covariate (no STI versus STI without diagnosis of distant progression versus STI after diagnosis of distant progression) because STI practices could have changed over the follow-up period. Finally, to determine if the performance of the BFRC schemes was sensitive to the method of calculating PSADT, the schemes were reconstructed and tested in unadjusted PCSM models based on PSAs over 12 months post BF.

Competing risks methodology was used to calculate the cumulative incidences of distant progression, STI and PCSM. Competing risks were defined as STI, and death for distant progression; death for STI; and death due to other or unknown cause for PCSM. Univariable analyses were performed to determine the cumulative incidences of these endpoints in the three strata of the best BFRC and were compared using Gray's test.

All analyses involving trial arms were conducted on an “intention to treat” basis and two-sided probability levels below 0.05 were considered significant. Analyses were performed using Stata Version 11.2.

## 3. Results

As on 31 August 2010, 485 (60.5%) out of the 802 eligible subjects had experienced biochemical failure (BF) prior to clinical failure or STI. Of these, 343 (71%) received STI, 150 (31%) died due to prostate cancer, and 69 (14%) died due to other causes. Median follow-up time after BF was 5.6 years (IQR 3.1–8.0).

### 3.1. Performance of Risk Strata Based on TTBF and PSADT

Table 1 summarises the performance of the 12 initial “range-finding” BFRC schemes in unadjusted models of PCSM after BF. The lowest c-indices were associated with schemes derived using cutpoints <9 months for PSADT or <3 years for TTBF to define the high risk categories, and >5 years for TTBF to define the low risk categories. Hence these cutpoints were not used to define the new “candidate” schemes. A total of 72 evaluable schemes were constructed according to our BFRC criteria using permutations of the following cutpoints: high risk PSADT (<3, <4, <5, <6 months), high risk TTBF (<1, <1.5, <2 years), low risk PSADT (>6, >9, >12, >18, >24 months), and low risk TTBF (>2, >3, >4 years).  Table 2 summarises the characteristics of 24 BFRC schemes based on the best three and worst three performing schemes for each high risk PSADT cutpoint. c-indices ranged between 0.685 and 0.732. The best schemes were characterized by a high risk category using a PSADT cutpoint <4 and <5 months and TTBF <1 year. These schemes had low risk categories with TTBF cutpoint >3 years and variable cutpoints for PSADT. The most predictive BFRC, with c-index of 0.732, was defined by high risk cutpoints PSADT <4 months and/or TTBF <1 year, and low risk cutpoints PSADT >9 months and/or TTBF >3 years. It divided subjects into three, sizeable risk groups with 246 (51%) categorised as low risk and 119 (25%) as high risk. Internal validation of the best BFRC model estimated the degree of overoptimism as 0.002, thus the optimism-corrected value of its performance was 0.730.

Sensitivity analyses using multivariable models confirmed that our findings were not influenced by potentially important confounding covariables (data not shown). The rankings remained stable across all models, with the best BFRC in the unadjusted model also being the most predictive scheme in the models adjusting for trial arm (c-index 0.747), as well as for prognostic factors known at time of BF (c-index 0.751). In addition, the best BFRC in the unadjusted model was also the most predictive (c-index 0.744) in schemes derived using 12 months of PSAs post-BF to calculate PSADT instead of 6 months of PSAs.

**Figure 1:**
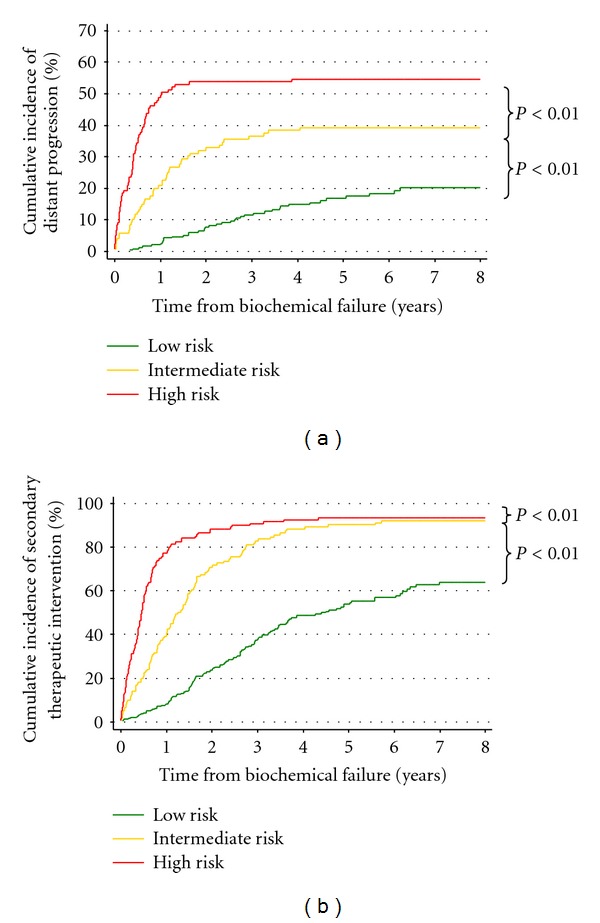
Outcomes after biochemical failure for the most predictive post-treatment failure category (BFRC) stratification scheme. (a) Cumulative incidence of distant progression. (b) Cumulative incidence of secondary therapeutic intervention.

**Figure 2:**
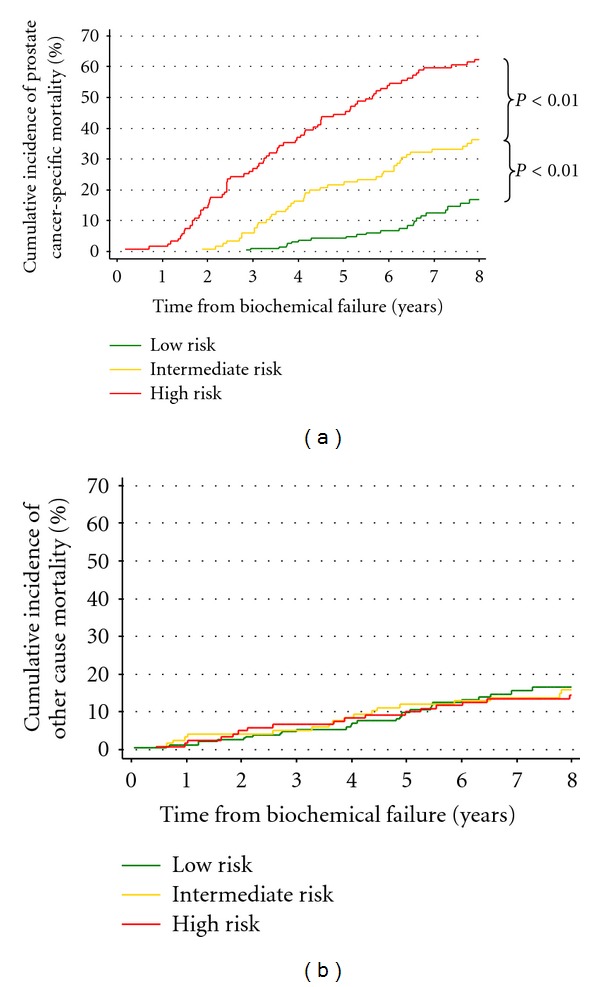
Mortality after biochemical failure for the most predictive post-treatment failure category (BFRC) stratification scheme. (a) Cumulative incidence of prostate cancer-specific mortality. (b) Cumulative incidence of other cause mortality.

Cumulative incidences of distant progression and STI for the best BFRC scheme are presented in Figures 1(a) and 1(b). The scheme separated the three risk categories very effectively. This figure shows that the majority of high risk subjects experienced distant progression and STI within the first 2 years after BF. In contrast, the cumulative incidences of distant progression and STI in low risk subjects 7 years after BF were approximately 20% and 60%, respectively. These findings were reflected in the cumulative incidences of PCSM shown in Figure 2(a). Pairwise comparisons of cumulative incidences were significant for all endpoints analysed (low versus intermediate risk, *P* < 0.01; intermediate versus high risk, *P* < 0.01).  Figure 2(b) shows that the cumulative incidence of death due to causes other than PC was near identical in all three risk categories, as would be hoped in an effective risk categorization scheme.

Table 3 presents pre- and postprimary treatment characteristics of the three risk categories of the best BFRC scheme. From this table, it can be shown that in the 59 subjects with tumours classified by the D'Amico stratification system [37] as intermediate risk, only 6.7% developed high risk BFs, whereas 76% developed low risk BFs. In contrast, 27% of the 426 subjects with D'Amico high risk tumours developed high risk BFs and 47% low risk BFs.

## 4. Discussion

This study has confirmed that TTBF and PSADT can be used to identify sizeable risk categories of men with poor, intermediate, and highly favourable outcomes after BF. The main strength of our study is that its findings are based on prospectively collected 10-year follow-up data from a randomised, clinical controlled trial. A further strength is the internal validation of the prognostic importance of the combination of the two variables. Our sensitivity analyses confirmed that the prognostic value of the combination was not influenced in multivariable models adjusting for treatment arm and other factors which could affect outcome, or in models using either 6 or 12 months of PSAs to estimate PSADT.

The optimal cutpoint ranges found to identify men at very high risk of early PCSM were PSADTs in the range <4 to <5 months and TTBF <1 year. These cutpoints were substantially lower than those found to be successful candidate surrogate endpoints for PCSM [17]. This finding is to be expected as the surrogate endpoints were derived from all eligible men on the trial (including those without BF) and measured PCSM from randomisation, whereas this study evaluated the subgroup of men who experienced BF after failing primary treatment and measured PCSM from BF.

The best risk scheme identified in this study had a modestly predictive optimism-corrected c-index of 0.730 for PCSM after BF. The high risk category for this scheme comprised men with PSADT <4 months and/or TTBF <1 year. Within a year of biochemical failure cumulative incidences of distant progression and STI for this category were 49% and 77%, respectively. In spite of the early introduction of STI, PCSM at 5 years after BF was 45%. These data suggest that many of these high risk men had microscopic metastases at the time of BF. Had modern imaging advances been available at the time, it is quite possible some of these men could have had imaging evidence of macroscopic metastastic disease. In either event, men in the high risk fail category could have benefited from immediate inclusion in trials of the new agents effective against CRPC, had they been available.

Although not the specific intention of this study, the low risk stratum in our optimal risk scheme identified a sizeable subgroup of men who could be safely reassured that their prognosis is good enough to avoid STI for many years and possibly indefinitely. The cutpoints identified in this subgroup were PSADT >9 months and/or TTBF >3 years. In these men cumulative incidences of distant progression and PCSM at 5 years were only 17% and 4%, respectively. At this time point cumulative incidence of death due to causes other than prostate cancer was 10%. This is a potentially important finding because two randomized trials designed to determine the value of early STI after biochemical failure [38, 39] were discontinued recently due to poor accrual.

For comparative purposes we have presented prognostically significant variables at biochemical failure identified in the most recently updated studies published since 2000 [5, 7–15, 40] (Table 4). In seven of these studies, Gleason score at or before treatment was used in determining a high risk stratum. In five of these, Gleason scores >7 were prognostic. All eleven studies used PSADT as a prognostic variable: three of these used PSADT cutpoints ≤12 months and one study used a PSADT cutpoint <9 months. Five examined TTBF but prognostic value for this variable was identified in only three studies. We found in our dataset that prognostically important variables prior to primary treatment, such as Gleason score, were no longer prognostic at the time of BF [16]. Amongst the explanations advanced we speculated that Gleason score based on prostatectomy findings would be more reliable in the present context than sextant fine needle biopsies or transurethral resection material. Five of the six studies in Table 4 where Gleason score was prognostic included sizeable numbers of men undergoing prostatectomy.  Table 5 shows the performance of the variables identified in these studies in predicting 5-year prostate cancer-specific mortality after biochemical failure in the TROG 96.01 dataset. The low prognostic value of pre-treatment Gleason score at biochemical failure in our dataset is illustrated by its failure to add prognostic value after substratification by PSADT or TTBF. The best stratification scheme from the studies presented in Table 5 had a c-index of 0.694 when modelled on TROG 96.01 data and was devised by Freedland et al. We attribute this to the use of PSADT >9 months and TTBF >3 years as cutpoints to define a low risk category group. It demonstrates that predictions based on prostatectomy data can be validated by a radiotherapy dataset based on men with locally advanced disease.

A few limitations of our study need to be acknowledged. Firstly, it is a secondary retrospective study not prespecified in the trial protocol. However, the disciplined prospective collection of data in the context of a randomized trial, as in this study, does avoid many of the unseen selection biases that exist in most retrospective clinical studies. Secondly, the radiation dose used in the trial (66 Gy) was low by modern standards [40]. Failure at the primary site would have been more frequent in the TROG 96.01 trial than it would be following the increased radiation doses used nowadays. In addition, distant progression as a result of metastasis from uncontrolled tumour at the primary site could also have been more common. It is quite probable that preventable local progressions (i.e., due to low radiation dosage) would have been associated with prolonged PSADTs (e.g., >10 months) and TTBFs (e.g., >3 years). If this is true then the major impact of the low doses used in the TROG 96.01 trial on our stratification scheme would be to increase the size of the low risk stratum. Thirdly, as pointed out earlier, modern imaging techniques could have indicated that some men in the high risk category already had macroscopic metastatic disease. They would therefore have M1 disease and arguably would not be considered high risk. They certainly would not be eligible for inclusion in trials where the first appearance of metastatic disease is the main trial endpoint. Fourthly, due to funding difficulties, there was no centralized review of histopathological material. However, although such a review could have increased the strong prognostic value of the assigned Gleason score at the time of randomization, we are very doubtful that it could have led to a large enough number of score reassignments to render this variable prognostic after BF. Finally, it has become a common practice in the clinic to commence STI shortly after BF. It is therefore possible that outcomes could be improved as a result of earlier intervention. However, in this dataset survival was shorter in men who received earlier STI [16] rendering this point moot.

Although we performed internal validation of our findings in this paper, external validation in a wider range of clinical scenarios is important. These datasets should comprise men with different initial risk profiles, and who have undergone a wider range of curative treatments than used in this study, for example, prostatectomy alone in earlier stage disease, and long-term AST and radiation in later stage disease. In recommending external validation, however, we need to caution that the proportion of men with low risk disease prior to primary treatment who develop “high risk” biochemical failures according to our definition is likely to be very small. In our dataset only 4 (6.8%) of 59 men with intermediate risk cancer who developed BFs were classified as high risk. In those with low risk disease treated by prostatectomy, other prognostic factors, such as Gleason score, margin status, seminal vesicle, or nodal involvement at prostatectomy, may assume greater prognostic importance and might need inclusion for a risk categorization scheme to be effective. We suspect therefore that men experiencing BF after radiation and long-term AST for LAPC will be most likely to derive benefits from a risk stratification based on PSADT and TTBF.

Finally, if risk stratification schemes based on TTBF and PSADT derived shortly after biochemical failure are to be reproducible, there is a need for international consensus on the most appropriate means of calculating PSADT within months of biochemical failure and ensuring that TTBF is measured accurately [17, 41]. The stratification schemes presented in this paper were produced with PSADTs calculated using the limited number of PSA values available within 6 months of BF in this dataset. Time will tell, however, whether the calculation of PSADT using at least four PSA values within 6 months of biochemical failure in the on-going RADAR trial run by our trials group [42] will produce more accurate estimates with improved prognostic precision.

## 5. Conclusions

This study has shown that time to biochemical failure and PSA doubling time can be combined to define risk stratification schemes after biochemical failure in men with locally advanced prostate cancer treated with short-term androgen suppression therapy and radiotherapy. External validation of these stratification schemes is necessary, particularly in datasets evaluating long-term androgen suppression therapy and radiotherapy.

## Figures and Tables

**Table 1:** Evaluation of 12 “range finding” post-biochemical failure risk categorization (BFRC) schemes in univariable competing risk models for prostate cancer-specific mortality (PCSM) after biochemical failure based on historical prognostic cutpoints for PSA doubling time and time to biochemical failure.

High risk^†^		Low risk^†^		BFRC performance			
PSADT	TTBF	PSADT	TTBF	c-index (95% CI)		Ranking^‡^	*P* value
<3	<1	>9	>3	0.724	(0.684–0.764)	1	—
<3	<2	>9	>4	0.705	(0.668–0.743)	4	0.095^§^
<3	<3	>9	>5	0.685	(0.651–0.719)	8	0.001
<3	<1	>12	>3	0.720	(0.680–0.760)	2	0.489^§^
<3	<2	>12	>4	0.695	(0.657–0.733)	5	0.026
<3	<3	>12	>5	0.667	(0.634–0.700)	9	<0.001
<6	<1	>15	>3	0.714	(0.680–0.749)	3	0.27^§^
<6	<2	>15	>4	0.691	(0.658–0.725)	6	0.033
<6	<3	>15	>5	0.659	(0.629–0.689)	11	<0.001
<9	<1	>18	>3	0.690	(0.656–0.723)	7	0.003
<9	<2	>18	>4	0.664	(0.633–0.695)	10	<0.001
<9	<3	>18	>5	0.630	(0.603–0.658)	12	<0.001

PSA: prostate-specific antigen; BFRC: biochemical failure risk categorization; PSADT: PSA doubling time (months); TTBF: time from biochemical (Phoenix) failure (years); CI: confidence interval; c-index: Harrell's concordance index.

^†^Risk is defined by PSADT and/or TTBF ranges specified.

^‡^Performance assessed by c-index, ranked highest (best) to lowest (worst). Performance against best BFRC compared using paired Student's *t*-test.

^§^c-index not significantly lower than best BFRC.

**Table 2:** Performance of 24 candidate post-biochemical failure risk categorization schemes (BFRC)* in univariable competing risk models for prostate cancer-specific mortality (PCSM) after biochemical failure.

BFRC model	High risk^†^					Low risk^†^				Intermediate risk^‡^			BFRC model performance^§^			
	*N*	PSADT	TTBF	HR	(95% CI)	*N*	PSADT	TTBF	HR	*N*	HR	(95% CI)	c-Index	(95% CI)	Rank	*P* value^#^
1	97	<3	<1	6.1	(3.8–9.8)	231	>9	>4	1	157	2.7	(1.8–4.3)	0.724	(0.685–0.763)	7	0.32
2	113	<3	<1.5	5.1	(3.3–7.7)	267	>6	>4	1	105	2.7	(1.8–4.2)	0.719	(0.678–0.760)	8	0.28
3	129	<3	<1.5	5.6	(3.5–9.0)	222	>9	>4	1	134	2.7	(1.7–4.3)	0.718	(0.679–0.757)	9	0.15
4	163	<3	<2	5.2	(3.2–8.5)	212	>9	>4	1	110	2.8	(1.6–4.7)	0.705	(0.668–0.743)	13	0.013
5	166	<3	<2	5.1	(3.2–8.3)	221	>12	>3	1	98	2.9	(1.7–4.9)	0.697	(0.658–0.736)	17	0.007
6	171	<3	<2	7.3	(3.9–13.7)	183	>12	>4	1	131	3.9	(2.1–7.5)	0.695	(0.657–0.733)	19	0.004
7	119	<4	<1	5.7	(3.7–8.8)	246	>9	>3	1	120	2.6	(1.7–4.2)	0.732	(0.695–0.769)	1^#^	—
8	119	<4	<1	7.0	(4.3–11.3)	225	>12	>3	1	141	3.1	(1.9–5.1)	0.730	(0.693–0.767)	2	0.70
9	120	<4	<1	8.2	(4.8–14.1)	208	>24	>3	1	157	3.5	(2.0–6.0)	0.729	(0.692–0.767)	3	0.68
10	188	<4	<2	8.1	(4.2–15.5)	180	>12	>4	1	117	3.7	(1.9–7.3)	0.705	(0.669–0.741)	14	0.019
11	196	<4	<2	7.4	(3.8–14.7)	163	>18	>4	1	126	2.9	(1.4–5.9)	0.697	(0.661–0.733)	16	0.005
12	196	<4	<2	8.0	(3.9–16.4)	159	>24	>4	1	130	3.1	(1.4–6.5)	0.697	(0.660–0.733)	18	0.005
13	150	<5	<1	7.6	(4.4–13.0)	201	>24	>3	1	134	3.4	(1.9–6.0)	0.727	(0.691–0.762)	4	0.55
14	149	<5	<1	5.2	(3.4–7.9)	239	>9	>3	1	97	2.5	(1.5–4.1)	0.726	(0.691–0.762)	5	0.41
15	149	<5	<1	6.3	(3.9–10.3)	218	>12	>3	1	118	3.1	(1.8–5.1)	0.726	(0.690–0.762)	6	0.45
16	201	<5	<2	8.4	(4.3–16.6)	173	>12	>4	1	111	3.9	(1.9–7.9)	0.700	(0.665–0.735)	15	0.007
17	209	<5	<2	7.8	(3.8–15.9)	156	>18	>4	1	120	3.0	(1.4–6.4)	0.693	(0.658–0.728)	20	0.002
18	209	<5	<2	8.5	(4.0–18.2)	152	>24	>4	1	124	3.2	(1.4–7.2)	0.693	(0.658–0.728)	21	0.002
19	141	<6	<1	5.0	(3.3–7.7)	235	>18	>2	1	109	2.4	(1.5–3.9)	0.714	(0.676–0.753)	10	0.18
20	184	<6	<1	6.8	(3.9–11.8)	190	>24	>3	1	111	2.7	(1.5–5.0)	0.713	(0.679–0.748)	11	0.07
21	140	<6	<1	4.7	(3.1–7.1)	242	>12	>2	1	103	2.4	(1.5–3.8)	0.712	(0.674–0.751)	12	0.12
22	220	<6	<2	7.7	(3.9–15.0)	167	>12	>4	1	98	3.3	(1.6–6.9)	0.691	(0.657–0.725)	22	0.001
23	228	<6	<2	7.0	(3.5–14.3)	150	>18	>4	1	107	2.4	(1.1–5.3)	0.685	(0.651–0.719)	23	<0.001
24	228	<6	<2	7.7	(3.6–16.4)	146	>24	>4	1	111	2.6	(1.1–6.0)	0.685	(0.651–0.719)	24	<0.001

PSA: prostate-specific antigen; BFRC: biochemical failure risk categorization; *N*: number of patients; PSADT: PSA doubling time (months); TTBF: time from biochemical (Phoenix) failure (years); HR: hazard ratio; CI: confidence interval; c-index: Harrell's concordance index.

*BFRC schemes presented include the best and worst three schemes for each high risk PSADT cutpoint from the 72 evaluable schemes.

^†^Risk is defined by PSADT and/or TTBF ranges specified.

^‡^PSADT and TTBF ranges are intermediate between the high and low risk ranges.

^§^Performance assessed by C-index, ranked highest (best) to lowest (worst). Performance against best BFRC compared using paired Student's *t*-test.

^#^The best BFRC scheme.

^¶^A *P*-value<0.05 for the paired Student's t-test indicates that the BFRC model is significantly worse (less predictive) than the best BRFC model.

**Table 3:** Pre- and post-treatment characteristics of the 485 subjects who developed biochemical (Phoenix) failure before clinical failure according to the most predictive post-biochemical failure risk category (BFRC) stratification scheme.

	Post-biochemical failure risk category					
	Low		Intermediate		High	
	(*n* = 246)		(*n* = 120)		(*n* = 119)	
Gleason score						
2–6	117 (47%)		36 (30%)		23 (19%)	
7	105 (43%)		53 (44%)		54 (45%)	
8–10	24 (10%)		31 (26%)		42 (35%)	

T stage						
T2b	73 (30%)		23 (19%)		16 (13%)	
T2c	87 (35%)		33 (28%)		36 (30%)	
T3,4	86 (35%)		64 (53%)		67 (56%)	

PSA (*μ*g/L)						
<10	45 (18%)		24 (20%)		20 (17%)	
≥10 and <20	102 (41%)		38 (32%)		24 (20%)	
≥20	99 (40%)		58 (48%)		75 (63%)	

Risk group*						
Intermediate	45 (18%)		10 (8%)		4 (3%)	
High	201 (82%)		110 (92%)		115 (97%)	

Primary treatment						
0 months AST	107 (44%)		45 (38%)		40 (34%)	
3 months AST	75 (30%)		35 (29%)		45 (38%)	
6 months AST	64 (26%)		40 (33%)		34 (28%)	

Age at BF (years)						
Median (range)	74 (54–89)		69 (54–88)		69 (44–81)	

PSADT (months)						
Median (IQR)	13.2 (9.4–19.8)		5.3 (4.3–6.4)		3.0 (1.9–3.8)	

TTBF (years)						
Median (IQR)	4.6 (3.4–7.3)		2.2 (1.5–2.7)		0.8 (0.5–1.4)	

STI						
No STI	121 (49%)		13 (11%)		8 (7%)	
STI without distant progression	89 (36%)		61 (51%)		47 (39%)	
STI with distant progression	36 (15%)		46 (38%)		64 (54%)	

*n*: number of subjects; PSA: prostate-specific antigen; AST: androgen suppression therapy; PSADT: PSA doubling time; TTBF: time from biochemical (Phoenix) failure; STI: secondary therapeutic intervention; IQR: interquartile range.

*D'Amico et al. risk classification.

**Table 4:** Eleven studies of prognostic factors after biochemical failure since 2000*.

Authors	Type of Series	Number in series	Endpoint	Pretreatment variables		Variables at biochemical failure		PSA at STI (*μ*g/L)	Other prognostic variables
				Gleason score	Clinical stage	TTBF years	PSADT months		
Sandler et al. (2000) [5]	Single hospital, radiotherapy, retrospective	154	Survival (PC specific and overall)	NS	NS	—	Continuous	—	—
D'Amico et al. (2002) [6]	Single hospital, radiotherapy, retrospective	381	Survival (PC specific)	—	—	NS	≤12	>10	—
Moul (2003) [7]^†^	Community database, prostatectomy	1352	Survival (overall)	>7	—	≤1	≤12	≤5, ≤10	—
Okotie et al. (2004) [8]	Single hospital, prostatectomy, retrospective	126	Metastases	—	—	—	<6	—	—
Stephenson et al. (2007) [9]	Multihospital, prostatectomy and radiotherapy, retrospective	1540	PSA progression	>7	Positive margins	—	≤10	Continuous	—
Freedland et al. (2005) [10]	Single hospital, prostatectomy, retrospective	379	Survival (PC specific)	>7	—	≤3	<3 3–8.9 ≥9	—	—
Dotan et al. (2005) [11]	Single hospital, prostatectomy, retrospective	239	Bone metastases	—	—	—	Continuous	Continuous (at time of scan)	—
Slovin et al. (2005) [12]	Single hospital, prostatectomy, retrospective	148	Metastases	>7	≥T3	—	Continuous	Continuous	—
Zhou et al. (2005) [13]	Community databases, prostatectomy and radiotherapy, retrospective	1159	Survival (PC specific)	>7 (radiotherapy alone)	—	NS	<3	—	—
Tollefson et al. (2007) [14]	Single hospital, prostatectomy, retrospective	1064	Survival (overall) metastases	—	—	—	<12 12–119.9 ≥120	—	—
Buyyounouski et al. (2008) [15]	Single hospital, radiotherapy, Retrospective	248	Survival (PC specific) Metastases	> 6 (metastases only)	—	<1.5	NS	—	PSA nadir <2 *μ*g/L (metastases only)

NS: nonsignificant; TTBF: time to biochemical failure; PSADT: PSA doubling time; STI: secondary therapeutic intervention; PC: prostate cancer.

*Only the most recent update of each series is included in the table.

^†^Review article.

**Table 5:** Predictive value of the prognostic stratification schemes in Table 4 using TROG 96.01 trial data with 5-year prostate cancer-specific mortality (± one standard error) as an endpoint.

Author	Variable	Cutpoints, mortality, number of patients				Cutpoints, Mortality, Number of patients				c-index*
	PSADT	≥6 months				<6 months				0.675
Okotie et al. [8]		8 ± 2%				37 ± 4%				
		*n* = 280 (58%)				*n* = 205 (42%)				

D'Amico et al. [6]	PSADT	≥12 months				<12 months				0.598
Tollefson et al. [14]		6 ± 2%				26 ± 3%				
		*n* = 138 (28%)				*n* = 347 (72%)				

	GS	≤7				>7				
	TTBF	>3 years		≤3 years		>3 years		≤3 years		0.694
Freedland et al. [10]^†^	PSADT	≥9 months	<9 months	≥9 months	<9 months	≥9 months	<9 months	≥9 months	<9 months	
		3 ± 2%	13 ± 5%	5 ± 4%	35 ± 4%	0%	0%	29 ± 17%	38 ± 6%	
		*n* = 139 (29%)	*n* = 57 (12%)	*n* = 41 (8%)	*n* = 151 (31%)	*N* = 14 (3%)	*N* = 7 (1%)	*N* = 7 (1%)	*N* = 69 (14%)	

	GS	≤7				>7				0.654
Zhou et al. [13]	PSADT	≥3 months		<3 months		≥3 months		<3 months		
		13 ± 2%		53 ± 8%		21 ± 5%		61 ± 12%		
		*n* = 340 (70%)		*n* = 48 (10%)		*n* = 76 (16%)		*n* = 21 (4%)		

Stephenson et al. [9]	GS	≤7				>7				0.647
	PSADT	>10 months		≤10 months		>10 months		≤10 months		
		4 ± 2%		27 ± 3%		11 ± 8%		34 ± 6%		
		*n* = 154 (32%)		*n* = 234 (48%)		*n* = 20 (4%)		*n* = 77 (16%)		

Buyyounouski et al. [15]	GS	≤6				>6				0.682
	TTBF	≥1.5 years		<1.5 years		≥1.5 years		<1.5 years		
		7 ± 2%		46 ± 9%		14 ± 3%		45 ± 5%		
		*n* = 143 (29%)		*n* = 33 (7%)		*n* = 213 (44%)		*n* = 96 (20%)		

*n*: number of TROG 96.01 subjects in study stratum; (*X*%): percentage of total number of TROG 96.01 subjects with biochemical failure (*n* = 485) in the study; GS: Gleason score; PSADT: PSA doubling time; TTBF: time to biochemical failure.

*Harrell's concordance index (higher c-index indicates that the model has a better predictive power).

^†^Due to subject numbers, subsets with PSADT <3 months and >15 months are not presented in the Freedland study.

## References

[1] Klotz LH (2006). PSA recurrence: definitions, PSA kinetics, and identifying patients at risk. *The Canadian Journal of Urology*.

[2] Moul JW (2000). Prostate specific antigen only progression of prostate cancer. *The Journal of Urology*.

[3] Simmons MN, Stephenson AJ, Klein EA (2007). Natural history of biochemical recurrence after radical prostatectomy: risk assessment for secondary therapy. *European Urology*.

[4] de Wit R (2006). New hope for patients with metastatic hormone-refractory prostate cancer. *European Urology Supplements*.

[5] Sandler HM, Dunn RL, McLaughlin PW, Hayman JA, Sullivan MA, Taylor JMG (2000). Overall survival after prostate-specific-antigen-detected recurrence following conformal radiation therapy. *International Journal of Radiation Oncology, Biology and Physics*.

[6] D’Amico AV, Cote K, Loffredo M, Renshaw AA, Schultz D (2002). Determinants of prostate cancer-specific survival after radiation therapy for patients with clinically localized prostate cancer. *Journal of Clinical Oncology*.

[7] Moul JW (2003). Variables in predicting survival based on treating “PSA-Only” relapse. *Urologic Oncology*.

[8] Okotie OT, Aronson WJ, Wieder JA (2004). Predictors of metastatic disease in men with biochemical failure following radical prostatectomy. *The Journal of Urology*.

[9] Stephenson AJ, Scardino PT, Kattan MW (2007). Predicting the outcome of salvage radiation therapy for recurrent prostate cancer after radical prostatectomy. *Journal of Clinical Oncology*.

[10] Freedland SJ, Humphreys EB, Mangold LA (2005). Risk of prostate cancer-specific mortality following biochemical recurrence after radical prostatectomy. *Journal of the American Medical Association*.

[11] Dotan ZA, Bianco FJ, Rabbani F (2005). Pattern of prostate-specific antigen (PSA) failure dictates the probability of a positive bone scan in patients with an increasing PSA after radical prostatectomy. *Journal of Clinical Oncology*.

[12] Slovin SF, Wilton AS, Heller G, Scher HI (2006). Time to detectable metastatic disease in patients with rising prostate-specific antigen values following surgery or radiation therapy. *Clinical Cancer Research*.

[13] Zhou P, Chen MH, McLeod D, Carroll PR, Moul JW, D’Amico AV (2005). Predictors of prostate cancer-specific mortality after radical prostatectomy or radiation therapy. *Journal of Clinical Oncology*.

[14] Tollefson MK, Slezak JM, Leibovich BC, Zincke H, Blute ML (2007). Stratification of patient risk based on prostate-specific antigen doubling time after radical retropubic prostatectomy. *Mayo Clinic Proceedings*.

[15] Buyyounouski MK, Hanlon AL, Horwitz EM, Pollack A (2008). Interval to biochemical failure highly prognostic for distant metastasis and prostate cancer-specific mortality after radiotherapy. *International Journal of Radiation Oncology, Biology and Physics*.

[16] Denham JW, Steigler A, Wilcox C (2009). Why are pretreatment prostate-specific antigen levels and biochemical recurrence poor predictors of prostate cancer survival?. *Cancer*.

[17] Denham JW, Steigler A, Wilcox C (2008). Time to biochemical failure and prostate-specific antigen doubling time as surrogates for prostate cancer-specific mortality: evidence from the TROG 96.01 randomised controlled trial. *The Lancet Oncology*.

[18] D’Amico AV, Moul JW, Carroll PR, Sun L, Lubeck D, Chen MH (2003). Surrogate end point for prostate cancer-specific mortality after radical prostatectomy or radiation therapy. *Journal of the National Cancer Institute*.

[19] Eastham JA (2005). Prostate-specific antigen doubling time as a prognostic marker in prostate cancer. *Nature Clinical Practice Urology*.

[20] Maffezzini M, Bossi A, Collette L (2007). Implications of prostate-specific antigen doubling time as indicator of failure after surgery or radiation therapy for prostate cancer. *European Urology*.

[21] Hanks GE, Hanlon AL, Lee WR, Slivjak A, Schultheiss TE (1996). Pretreatment prostate-specific antigen doubling times: clinical utility of this predictor of prostate cancer behavior. *International Journal of Radiation Oncology, Biology and Physics*.

[22] Ward JF, Blute ML, Slezak J, Bergstralh EJ, Zincke H (2003). The long-term clinical impact of biochemical recurrence of prostate cancer 5 or more years after radical prostatectomy. *The Journal of Urology*.

[23] Albertsen PC, Hanley JA, Penson DF, Fine J (2004). Validation of increasing prostate specific antigen as a predictor of prostate cancer death after treatment of localized prostate cancer with surgery or radiation. *The Journal of Urology*.

[24] Pound CR, Partin AW, Eisenberger MA, Chan DW, Pearson JD, Walsh PC (1999). Natural history of progression after PSA elevation following radical prostatectomy. *Journal of the American Medical Association*.

[25] Pollack A, Hanlon AL, Movsas B, Hanks GE, Uzzo R, Horwitz EM (2003). Biochemical failure as a determinant of distant metastasis and death in prostate cancer treated with radiotherapy. *International Journal of Radiation Oncology, Biology and Physics*.

[26] Zelefsky MJ, Ben-Porat L, Scher HI (2005). Outcome predictors for the increasing PSA state after definitive external-beam radiotherapy for prostate cancer. *Journal of Clinical Oncology*.

[27] Alcántara P, Hanlon A, Buyyounouski MK, Horwitz EM, Pollack A (2007). Prostate-specific antigen nadir within 12 months of prostate cancer radiotherapy predicts metastatis and death. *Cancer*.

[28] Freedland SJ, Humphreys EB, Mangold LA, Eisenberger M, Partin AW (2006). Time to prostate specific antigen recurrence after radical prostatectomy and risk of prostate cancer specific mortality. *The Journal of Urology*.

[29] Zagars GK, Pollack A (1997). Kinetics of serum prostate-specific antigen after external beam radiation for clinically localized prostate cancer. *Radiotherapy and Oncology*.

[30] Hanks GE, D’Amico A, Epstein BE, Schultheiss TE (1993). Prostatic-specific antigen doubling times in patients with prostate cancer: a potentially useful reflection of tumor doubling time. *International Journal of Radiation Oncology, Biology and Physics*.

[31] Sartor CI, Strawderman MH, Lin XH, Kish KE, McLaughlin PW, Sandler HM (1997). Rate of PSA rise predicts metastatic versus local recurrence after definitive radiotherapy. *International Journal of Radiation Oncology, Biology and Physics*.

[32] Denham JW, Steigler A, Lamb DS (2005). Short-term androgen deprivation and radiotherapy for locally advanced prostate cancer: results from the Trans-Tasman Radiation Oncology Group 96.01 randomised controlled trial. *The Lancet Oncology*.

[33] Roach M, Hanks G, Thames H (2006). Defining biochemical failure following radiotherapy with or without hormonal therapy in men with clinically localized prostate cancer: recommendations of the RTOG-ASTRO Phoenix Consensus Conference. *International Journal of Radiation Oncology, Biology and Physics*.

[34] Denham JW, Steigler A, Lamb DS (2011). Short-term neoadjuvant androgen deprivation and radiotherapy for locally advanced prostate cancer: 10-year data from the TROG 96.01 randomised trial. *The Lancet Oncology*.

[35] Fine JP, Gray RJ (1999). A proportional hazards model for the subdistribution of a competing risk. *Journal of the American Statistical Association*.

[36] Harrell FE, Lee KL, Mark DB (1996). Multivariable prognostic models: issues in developing models, evaluating assumptions and adequacy, and measuring and reducing errors. *Statistics in Medicine*.

[37] D’Amico AV, Whittington R, Malkowicz SB (1998). Biochemical outcome after radical prostatectomy, external beam radiation therapy, or interstitial radiation therapy for clinically localized prostate cancer. *The Journal of the American Medical Association*.

[38] NCT00110162.

[39] ELAAT Trial Early vs late androgen ablation therapy for recurrent prostate cancer post-radiotherapy. http://www.clinicaltrials.gov/ct/show/NCT00439751.

[40] Dearnaley DP, Sydes MR, Graham JD (2007). Escalated-dose versus standard-dose conformal radiotherapy in prostate cancer: first results from the MRC RT01 randomised controlled trial. *The Lancet Oncology*.

[41] Denham JW, Kumar M, Gleeson PS (2009). Recognizing false biochemical failure calls after radiation with or without neo-adjuvant androgen deprivation for prostate cancer. *International Journal of Radiation Oncology, Biology and Physics*.

[42] http://clinicaltrials.gov/ct/show/NCT00193856?order=5.

